# A novel high light-inducible carotenoid-binding protein complex in the thylakoid membranes of *Synechocystis* PCC 6803

**DOI:** 10.1038/srep09480

**Published:** 2015-03-30

**Authors:** Soumana Daddy, Jiao Zhan, Saowarath Jantaro, Chenliu He, Qingfang He, Qiang Wang

**Affiliations:** 1 Key Laboratory of Algal Biology, Institute of Hydrobiology, Chinese Academy of Sciences, Wuhan 430072, China; 2 University of Chinese Academy of Sciences, Beijing 100039, China; 3 Department of Biology, University of Arkansas at Little Rock, Little Rock, AR 72204; 4 Biotechnology Research Center, Shandong Academy of Agricultural Sciences, Jinan, Shandong 250100, China; 5 Department of Biochemistry, Faculty of Science, Chulalongkorn University, Payathai Road, Patumwan, Bangkok 10330, Thailand

## Abstract

*Synechocystis* sp. PCC 6803 is a model cyanobacterium extensively used to study photosynthesis. Here we reveal a novel high light-inducible carotenoid-binding protein complex (HLCC) in the thylakoid membranes of *Synechocystis* PCC 6803 cells exposed to high intensity light. Zeaxanthin and myxoxanthophyll accounted for 29.8% and 54.8%, respectively, of the carotenoids bound to the complex. Using Blue-Native PAGE followed by 2D SDS-PAGE and mass spectrometry, we showed that the HLCC consisted of Slr1128, IsiA, PsaD, and HliA/B. We confirmed these findings by SEAD fluorescence cross-linking and anti-PsaD immuno-coprecipitation analyses. The expression of genes encoding the protein components of the HLCC was enhanced by high light illumination and artificial oxidative stress. Deletion of these proteins resulted in impaired state transition and increased sensitivity to oxidative and/or high light stress, as indicated by increased membrane peroxidation. Therefore, the HLCC protects thylakoid membranes from extensive photooxidative damage, likely via a mechanism involving state transition.

Exposure to high-intensity light (HL) adversely affects the photosynthetic performance, cell growth, and viability of photosynthetic organisms. The damage is largely attributed to oxygen-dependent destruction of the photosynthetic apparatus and other cellular components^1^,^2^.

Oxygenic photosynthetic organisms synthesize stress-associated proteins during exposure to HL. These proteins are often important for the acclimation of cells to HL. A family of HL-inducible genes, called *hli* or *scp* genes^3^,^4^, encoding high light-inducible polypeptides (HLIP) with similarity to the light-harvesting chlorophyll a/b-binding proteins (LHCP) of plants, was shown to be critical for the survival of cyanobacteria under HL conditions^5^. All four HLIPs in *Synechocystis* PCC 6803, i.e., HliA, B, C, and D, are associated with photosystem I (PSI), and stabilize PSI trimers specifically under HL conditions. HliA and HliB also interact with Slr1128^6^. Another stress-associated protein, the iron stress-inducible chlorophyll-binding protein IsiA, stabilizes PSI trimers under HL conditions. Interestingly, IsiA, which is expressed at low levels under normal growth conditions and is induced by both HL and oxidative stress, is critical for the formation of PSI trimers in low-intensity light^7^. This is in contrast to the expression of PsaL and PsaI, both of which are essential for the formation of PSI trimers^8^, but are not strongly modified by stress conditions as compared to IsiA or HLIPs.

Even though the PSI complexes of green plants and cyanobacteria have similar structures and carry out similar functions, i.e., mediating the electron transfer between luminal plastocyanin (or cytochrome *c*_6_) and stromal ferredoxin (Fd; or flavodoxin), there are several differences between them. One of the differences is that higher plants contain only monomeric PSI, whereas cyanobacteria harbor both monomeric and trimeric PSI^9^,^10^,^11^. It is unclear why PSI trimers exist in cyanobacteria; however, studies in *Spirulina platensis* provided some insight into this phenomenon. In this cyanobacterium, the absorption spectra of the chlorophyll (Chl) components of the PSI trimers and monomers differ^12^; only the PSI trimers contain the extremely red-shifted Chl (the so-called red chlorophyll), which absorbs at 735 nm and gives rise to a 760-nm fluorescence emission peak (F760) at 77 K. The PSI monomer shows emission peaks only at 725–730 nm under the same conditions. In addition, the 760-nm emission band is only visible under reducing conditions, directly reflecting the redox state of P700^12^,^13^. Red Chl is thought to funnel light energy to P700^14^,^15^,^16^, thereby increasing the cross-section of light absorption, or to dissipate excess energy into heat, thereby protecting PSI against photodestruction^17^.

The orange carotenoid protein (OCP), a 35-kDa two-domain soluble protein, was shown to trigger fluorescence quenching of phycobilisome (PBS) under blue-green light illumination^18^, and was proposed to be a photoactive protein that senses light intensity and triggers photoprotection^19^. The protein, which was first discovered by Holt and Krogman^20^, contains one non-covalently bound carotenoid molecule^20^,^21^,^22^,^23^,^24^. While the OCP is conserved among cyanobacteria except *Prochlorococci*^21^, the carotenoid differs in different species, being zeaxanthin in *Anacystis nidulans* and *Lyngbya wholei*^25^, 3′-hydroxyechinenone in *Arthrospira maxima*, and a glycoside derivative of 3′-hydroxyechinenone in *Synechocystis* PCC 6803^21^,^23^,^24^,^26^. Water-soluble OCP may function as a dimer in cells^22^,^26^.

Here, we report the discovery of a novel high light-inducible carotenoid-binding protein complex (HLCC). This complex is normally concealed by trimeric PSI on a sucrose gradient, but was readily detected in a *Synechocystis* strain lacking PsaL (i.e., ΔPsaL) and hence lacking PSI trimers. The HLCC contains Slr1128, IsiA, PsaD, and HliA/B as its intrinsic protein components, binds zeaxanthin and myxothanxophyll, and is critical for the survival of cyanobacterial cells in HL conditions.

## Results

### Induction of a novel carotenoid-binding membrane protein complex by HL treatment

**Figure 1:**
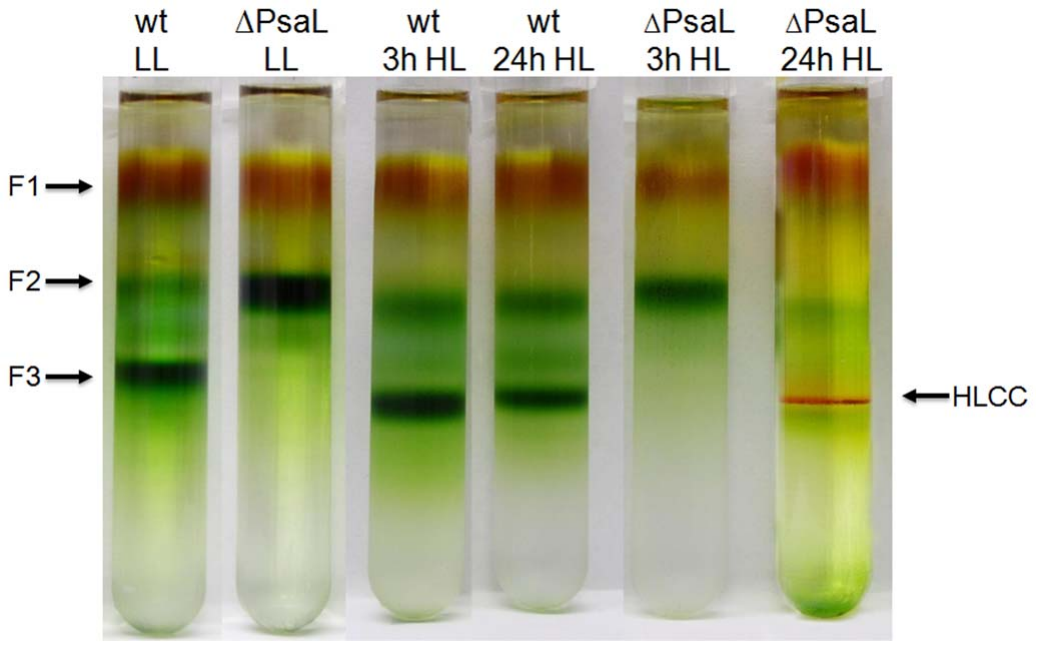
Sucrose gradient fractions of thylakoid protein complexes from wild-type and ΔPsaL *Synechocystis* PCC 6803 strains. Thylakoid membranes were isolated from wild-type and PsaL deletion strains grown in LL or HL for 3 h and 24 h. Thylakoid protein complexes were separated by step sucrose gradient ultracentrifugation. Fractions are as indicated.

Thylakoid membranes were isolated from *Synechocystis* PCC 6803 cells grown in LL or HL, solubilized in mild detergent, and fractionated by sucrose gradient ultra-centrifugation (Fig. 1). Three pigmented fractions were detected in the samples of wild-type cells grown in LL. The uppermost orange fraction, F1, contained mainly free pigments; the middle green fraction, F2, contained PSI and PSII monomers; and the lower dark green fraction, F3, contained PSI trimers^6^,^27^. In agreement with previous reports^28^, the ΔPsaL strain grown in LL contained only F1 and F2, and lacked PSI trimers. Interestingly, when ΔPsaL cells were grown in HL for 24 h, an orange protein complex appeared at the gradient position of F3. The orange color of this membrane protein fraction indicates the presence of carotenoids.

**Figure 2:**
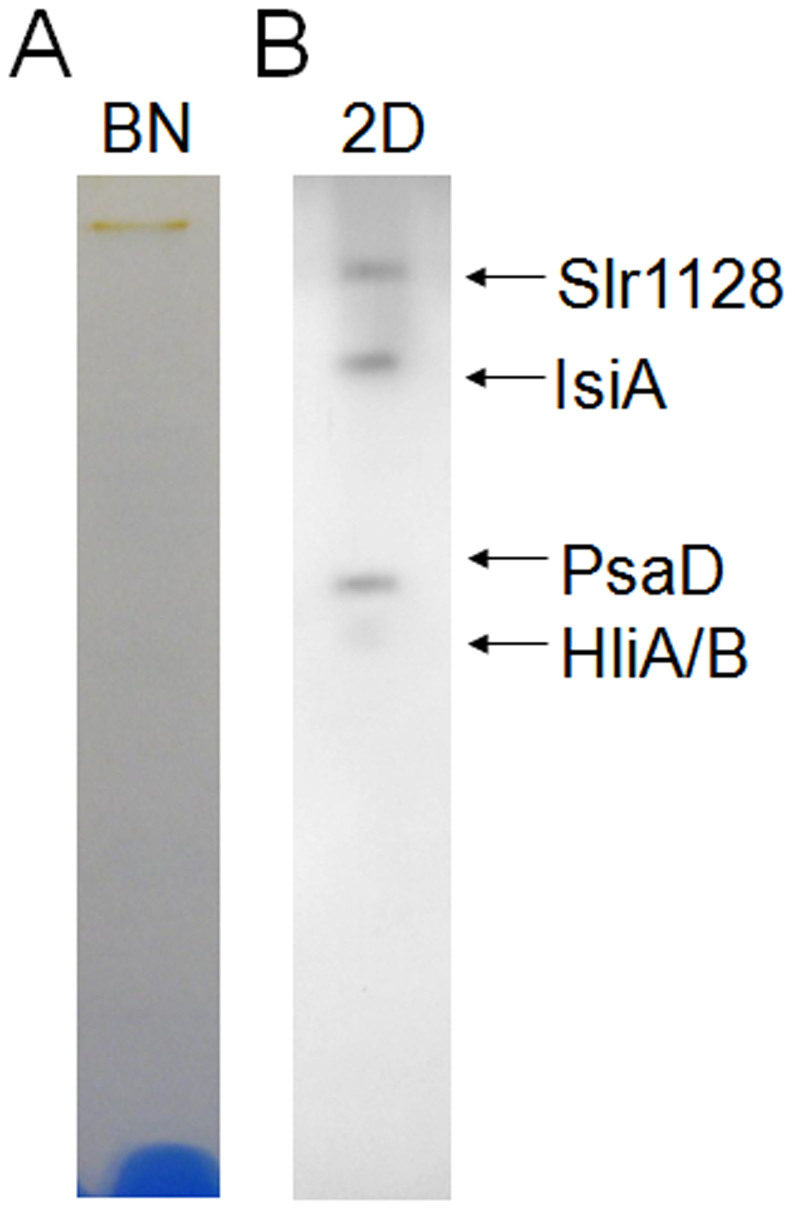
Separation of the HLCC by Blue-Native PAGE followed by second dimension Tricine-SDS-PAGE. The HLCC fraction was carefully collected from the sucrose gradient and separated by BN PAGE (A), and the resulting single band was excised and denatured in 1.5 X SDS sample buffer and further separated on a 12–20% Tricine-SDS gel with 6 M urea (B). The proteins were visualized by silver staining and identified by mass spectrometry.

We further purified the fraction by Blue-Native PAGE. As shown in Fig. 2A, a single orange band was detected, indicating that there is one carotenoid-containing protein complex in the sucrose gradient fraction. We named this orange protein complex high light-inducible carotenoid-binding complex (HLCC). The orange Blue-Native band was then excised, denatured, and separated on a Tricine-SDS-PAGE gel (Fig. 2B), and four protein bands were revealed in the complex by silver staining. The bands were excised and identified by mass spectrometry. The three larger bands (from largest to smallest) contained the hypothetical protein Slr1128, the iron stress-induced chlorophyll-binding protein IsiA, and PsaD, a subunit of PSI, respectively. The fourth band at the lower end of the gel was identified as HliA and HliB.

**Figure 3:**
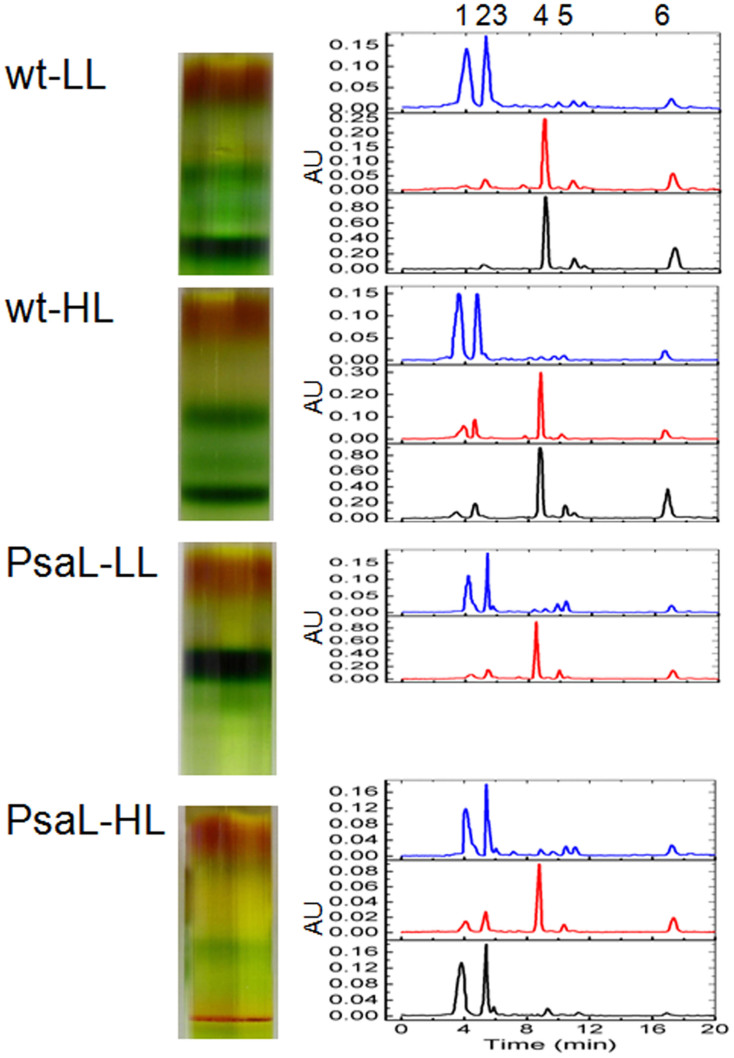
HPLC separation of the pigments in the sucrose gradient fractions. Pigments were identified by comparing their retention times and absorption spectra as described previously (Takaichi et al., 2001). Peaks are 1, myxoxanphyll; 2, zeaxanthin; 3, hydroxyechinenone; 4, chlorophyll a; 5, echinenone; and 6, β-carotene.

To determine the pigment composition of the complex, the pigments were extracted and separated by HPLC according to^29^. As shown in Fig. 3, myxoxanthophyll and zeaxanthin accounted for most of the pigment present in the HLCC, and there was a negligible amount of Chl a.

### Slr1128, IsiA, PsaD, and HliA/B are intrinsic components of the HLCC

**Figure 4:**
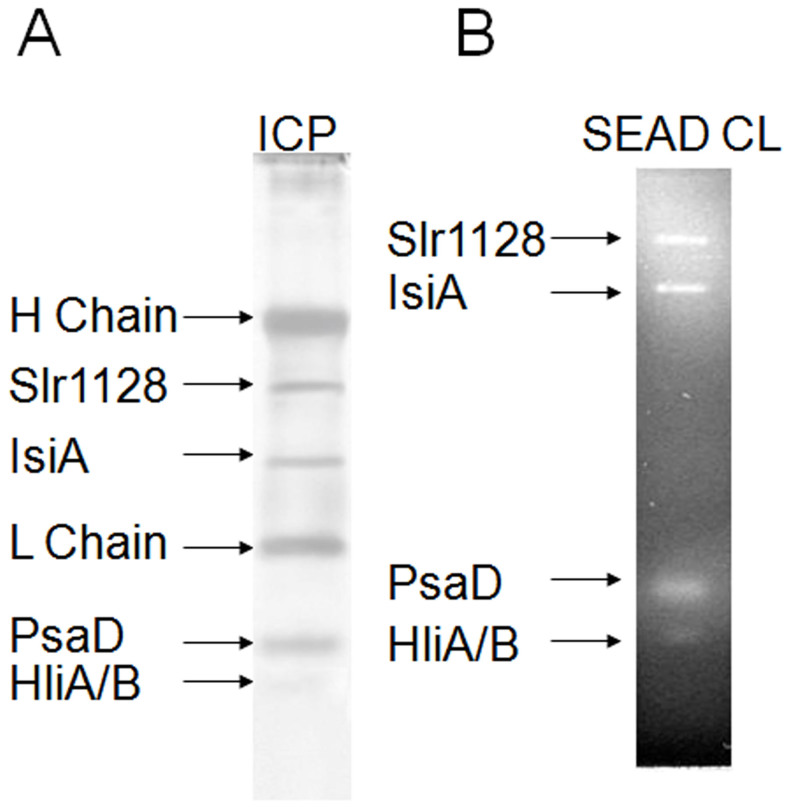
Immuno-coprecipitation and SEAD crosslinkage of the HLCC. The HLCC collected from the sucrose gradient was (A) incubated with anti-PsaD and then Protein G resin, and the eluted proteins were separated by SDS PAGE, and (B) allowed to react in the dark with SEAD, quenched with lysine, photoactivated with UV light, and separated by SDS PAGE.

To characterize the HLCC further, we investigated whether IsiA, PsaD, and HliA/B are intrinsic components of the complex using anti-PsaD immuno-coprecipitation. Briefly, the sucrose gradient fraction containing the HLCC was collected and incubated first with anti-PsaD antibody and subsequently with protein G Sepharose beads. The beads were then loaded on an empty column and the protein complex was eluted and separated by SDS-PAGE. In addition to the heavy and light chains of the antibody, the anti-PsaD antibody identified Slr1128, IsiA, PsaD, and HliA/B as the major components of the HLCC (Fig. 4a).

We then confirmed these results by crosslinking analysis using SEAD (sulfosuccinimidyl-2(7-azido-4-methylcoumarin-3-acetamido)-ethyl-1,3'- dithiopropionate), a crosslinker with a fluorescent spacer arm that fluoresces brightly when the disulfide bond between the crosslinked proteins is cleaved. Briefly, the non-covalent protein complex was collected from F3 of the sucrose gradient, and the crosslinker was covalently incorporated into the complex by chemical bonding to free amino groups, mainly from lysine residues. The proteins of the complex were subsequently crosslinked by a photochemical reaction of the crosslinker's azido group. Cleavage of the disulfide group in the crosslinker by reduction released the fluorescently labeled proteins and removed the fluorescent group from proteins that were not cross-linked. The fluorescently labeled components of the protein complex were then separated by SDS-PAGE and detected by fluorescence imaging. As shown in Fig. 4B, when crosslinked with SEAD and separated by SDS-PAGE, the HLCC complex shows four fluorescent bands under UV light, which correspond to Slr1128, IsiA, PsaD, and HliA/B, further demonstrating that these proteins are intrinsic components of the HLCC.

### The HLCC is induced by high-intensity light, iron starvation, and oxidative stress

**Figure 5:**
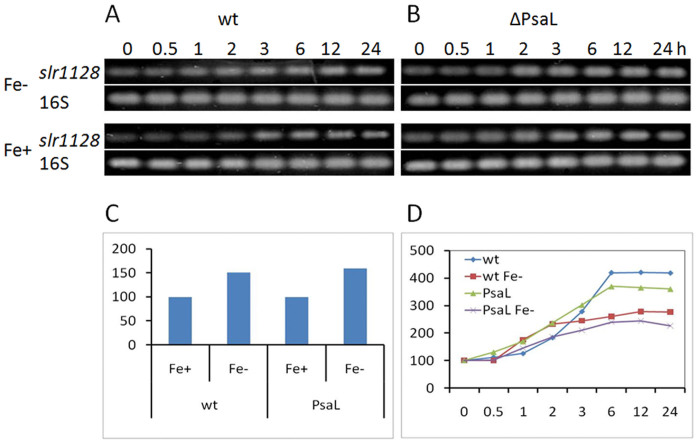
Semi-quantitative PCR analysis of the induction of *slr1128* transcript during HL treatment in the presence and absence of iron. (A) Wild-type and (B) ΔPsaL *Synechocystis* strains were cultured in iron-deplete (Fe-) of iron-replete (Fe+) BG11 media to the mid-logarithmic growth phase (OD_730_ ~ 0.8 after 48 hours), diluted with fresh medium to OD_730_ ~ 0.1, and exposed to HL at 30°C. Samples for semi-quantitative PCR were taken at various time points as indicated; (C), Gel Bands quantification at time 0 as standardized with 16S; (D), Gel Bands quantification of (A) and (B), time 0 were set to 100 for easier comparation.

We previously showed that one of the major components of the HLCC discovered in the current study, IsiA, could be induced by iron depletion, HL stress, and rose bangal (RB) treatment^7^. To study the transcript induction of the other major component of the HLCC, i.e., Slr1128, we cultured both the wild-type and ΔPsaL strains in BG11, with or without iron, to the mid-logarithmic growth phase (OD_730_ ~ 0.8), and then diluted the cultures to OD_730_ ~ 0.1 with fresh medium and subjected them to HL. Samples were taken at various time points for semi-quantitative RT-PCR analysis to examine the expression level of *slr1128* under various growth conditions. As shown in Fig. 5, *slr1128* is expressed in both the wild-type (Fig. 5A) and ΔPsaL (Fig. 5B) strains under standard conditions, and is further induced by HL and iron depletion (Fig. 5C). Expression levels plateaued after a 6-hour induction (Fig. 5D).

**Figure 6:**
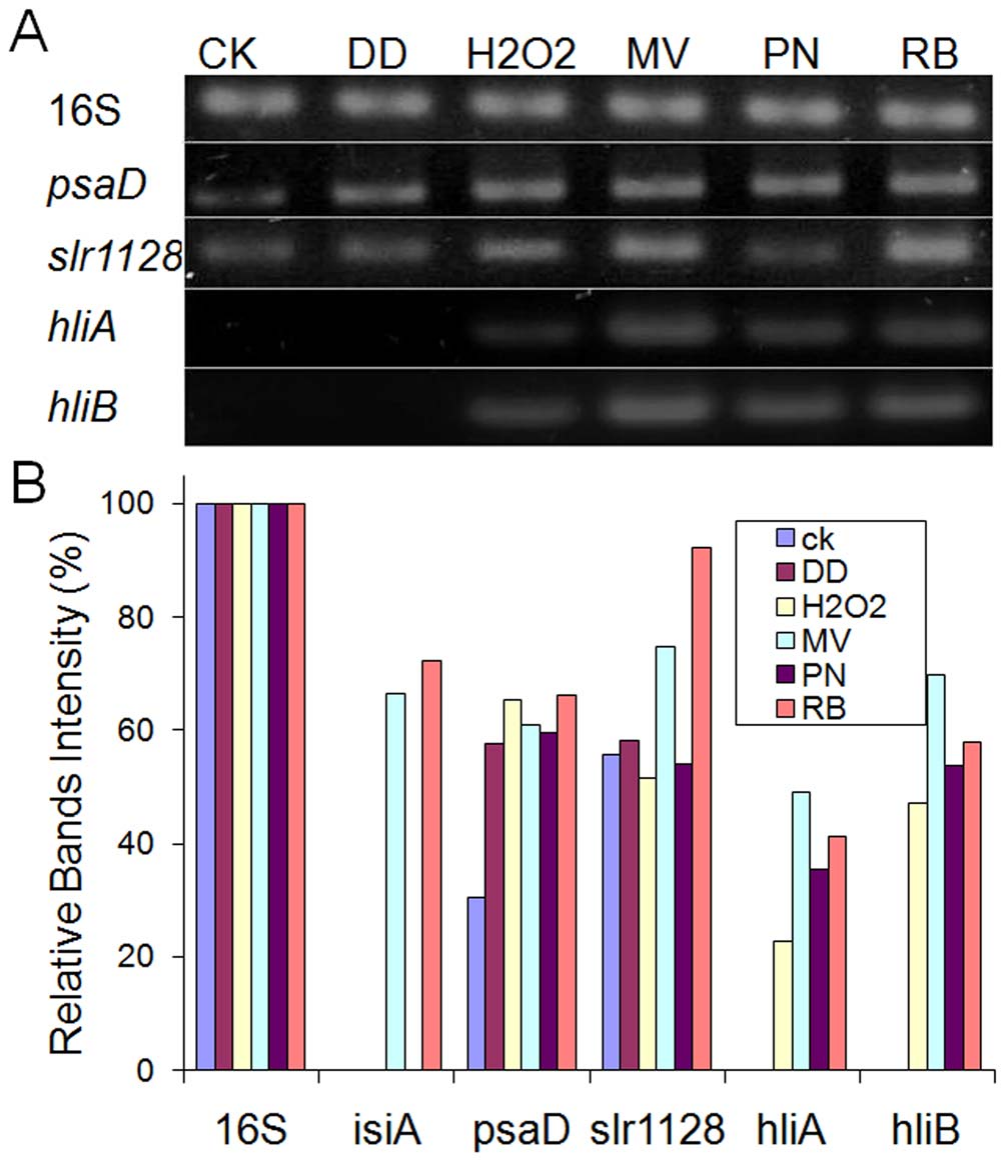
Semi-quantitative PCR analysis of genes encoding components of the HLCC upon 12 hours of exposure to chemically-induced oxidative stress. (A) Transcript levels upon treatment with the indicated artificial oxidants, and (B) quantification of the transcript levels shown in (A).

To further identify the conditions that induce transcription of the genes encoding components of the HLCC, we created artificial oxidative stress environments using the simple peroxide, hydrogen peroxide (H_2_O_2_); the lipid peroxide, decadienal (DD); the nitrite oxidant, peroxynitrite (PN); the redox-active compound, methyl viologen (MV); and the singlet oxygen generator, rose Bengal (RB). Fig. 6 shows that after a 12-hour treatment, *psaD* was strongly induced by all chemicals tested. Both *hliA* and *hliB*, which are normally not transcribed, were induced by H_2_O_2_, MV, PN, and RB. Furthermore, *slr1128* was induced by H_2_O_2_, MV, and RB. By contrast, as presented in our previous study, *isiA* was only induced by MV and RB^7^.

### Deletion of slr1128 resulted in increased sensitivity to oxidative stress due to membrane peroxidation

**Figure 7:**
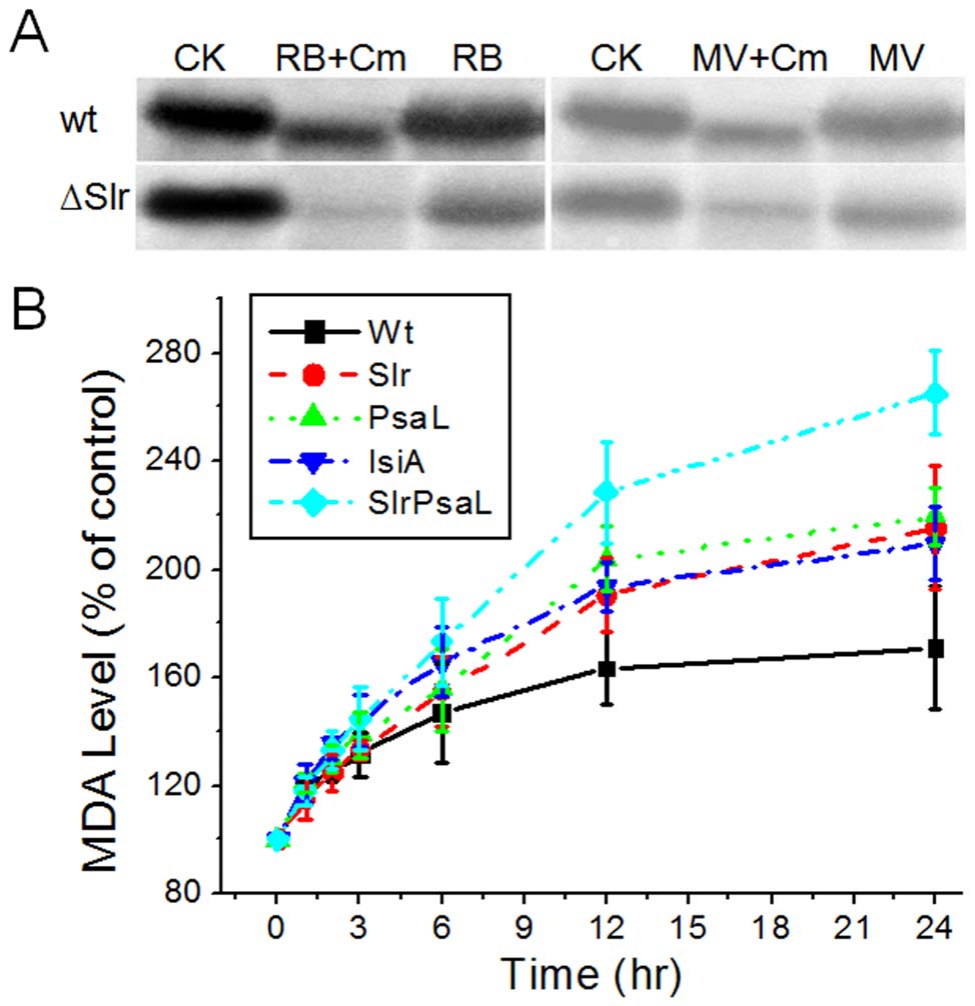
Sensitivity of the HLCC mutants to oxidative stress. (A) D1 protein level in RB- and MV-treated cells in the presence (+Cm) or absence of chloramphenicol. Wild-type (Wt) and ΔSlr1128 (ΔSlr) cells in the mid-logarithmic growth phase (OD730, 0.6–0.8) were diluted to an OD_730_ of 0.1 before exposure to HL for 12 h. Blots of the thylakoid membrane proteins were probed with polyclonal anti-D1 antibodies. (B) The level of lipid peroxidation in the HLCC mutant strains during HL treatment. Curves were generated by averaging the data obtained from three representative experiments.

As shown above, all components of the HLCC were induced by oxidative stress (Fig. 6). We previously showed that PsaL, Slr1128, and IsiA are all important for *Synechocystis* survival upon exposure to HL^6^,^7^. To evaluate the function of Slr1128 in oxidative stress conditions, we treated the deletion strain ΔSlr1128 and the wild type with RB and MV in the presence and absence of the chloroplast protein synthesis inhibitor chloramphenicol (Cm) for 12 h. We then measured the D1 protein level by immunoblot analysis as an indicator of oxidative stress. The ΔSlr1128 mutant strain was more sensitive than the wild type to both RB and MV treatment (Fig. 7), as indicated by the ~50% reduction in D1 protein after the treatment, and this sensitivity was enhanced in the presence of Cm (~70% reduction in D1). These results suggest a role for Slr1128 in the response to oxidative stress.

To determine whether the enhanced light sensitivity of the ΔSlr1128 strain is due to HL-induced oxidative stress, we monitored the level of malondialdehyde (MDA), a product of lipid peroxidation, during HL treatment. The MDA level of all strains increased at the start of HL stress, and plateaued after 12 h of exposure to HL (Fig. 7B). After a 24-h HL treatment, MDA levels in the ΔSlr1128/ΔPsaL double mutant increased by 160%, and the levels in all of the single mutants increased by about 110%, while those of the wild type increased by only 70%, indicating that the rate of membrane peroxidation was greater in the mutant than in the wild type under HL conditions. Furthermore, mutation of both *slr1128* and *psaL* had a greater effect than mutation of either gene alone, suggesting that SLR1128 and PsaL act synergistically. Thus, SLR1128 and PsaL play important roles in HL/oxidative stress.

### Deletion of slr1128 resulted in impaired state transition

**Figure 8:**
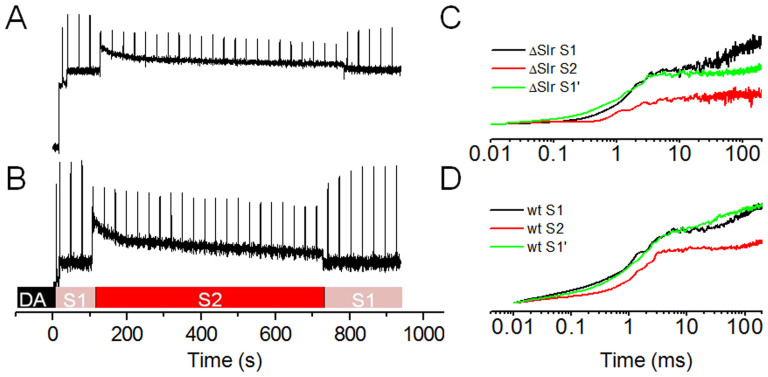
State transition of the wild-type and ΔSlr1128 strains. Representative traces of state transition analysis of the ΔSlr1128 (A) and wild-type (B) strains. Fast fluorescence induction kinetics of the ΔSlr1128 (C) and wild-type (D) strains at state 1 (S1), state 2 (S2), and state transition level (S′).

State transition is a physiological adaptation in cyanobacteria that balances the distribution of light energy absorbed by phycobilisomes between PSI and PSII^30^,^31^. Prompted by the previous observation that deletion of *isiA* altered the state transition capacity^7^, we examined whether state transition was also altered in the ΔSlr1128 strain. As shown in Fig. 8, the wild type has a fully functional state transition, as indicated by the fast and full relaxation of maximum fluorescence after high actinic light illumination (Fig. 8B and D). Therefore, the photoinhibitory effect of HL (400 μmol photon m^−2^ s^−1^) on the wild type is negligible. By contrast, the ΔSlr1128 mutant exhibited a very low state transition level and resulted in substantial photoinhibitory quenching, as indicated by its poor recovery of maximum fluorescence (Fig. 8A and C).

## Discussion

In the current study, we report the discovery of the novel high light-inducible carotenoid-binding protein complex (HLCC), which is normally concealed by trimeric PSI on a sucrose gradient, in the ΔPsaL strain of *Synechocystis* PCC 6803 subjected to HL stress. The HLCC contains Slr1128, IsiA, PsaD, and HliA/B as its intrinsic components, and binds to zeaxanthin and myxothanxophyll.

Slr1128 is a hypothetical protein highly conserved in cyanobacteria; the amino acid sequence identity between homologs of this protein in various species of cyanobacteria is generally above 80%. It also appears to be present in plants, with homologs in plants sharing 59–66% amino acid sequence identity with cyanobacterial Slr1128. Slr1128 has a transmembrane domain at its N-terminus and is likely an integral membrane protein, as it was also identified as a thylakoid membrane protein by other researchers^32^. Furthermore, Slr1128 exhibits high levels of similarity (around 35% amino acid sequence identity) to human and animal stomatins, which are thought to regulate an associated ion channel, and to a bacterial protease (HflC), but the similarity between Slr1128 and stomatins or HflC is not as high as that between the counterparts of these proteins in other cyanobacteria or plants. We previously reported that Slr1128 was associated with HliA/B, and suggested that it functions in photoprotection^6^. Here, we found that Slr1128 was also present in the HLCC, and appears to be closely associated with the HL-inducible proteins, HliA and HliB. This latter finding corresponds well with our previous work^6^.

The peripheral PsaD subunit is located at the stromal side of photosystem I^33^, and is highly conserved in all photosynthetic organisms (including bacteria with Fe-S-type reaction centers). With PsaL, PsaD plays a critical role in the assembly and stability of PSI^34^,^35^,^36^ and mediates Fd electrostatic guidance and docking on PSI^33^,^37^,^38^,^39^,^40^,^41^. Superoxide is formed by Fd at PSI through the Mehler reaction^42^.

In this study, we showed that PsaD, Slr1128, IsiA, and HliA/B formed a novel protein complex (the HLCC) in the cyanobacterium *Synechocystis* PCC 6803. Our earlier results showed that HliA and HliB stabilize PSI trimers, interact with Slr1128, and protect cells under HL conditions^6^. As our recent findings also showed that IsiA, which was identified here as one of the major components of the HLCC, forms a supercomplex with PSI and PSII, IsiA is important both for maintaining trimeric PSI integrity and for acclimation to HL and may protect cyanobacteria from HL and oxidative stresses through state transition^7^. Interestingly, we now found that the other major component of the HLCC, Slr1128, was also important for the response to oxidative stresses and state transition (Fig. 7 and 8). These results suggest that Slr1128 interacts with IsiA to form the HLCC and protects cyanobacteria from oxidative stresses by mediating state transitions.

Thus, we propose that, by interacting with the peripheral PSI protein PsaD, which provides a docking site for Fd and interacts with PsaB, PsaC, PsaE, and PsaL, the HLCC complex stabilizes trimeric PSI and protects the photosystems, especially PSI, from HL and oxidative stresses, probably through (1) direct scavenging of the superoxide and/or other reactive oxygen species (ROS) produced at Fd; and (2) mediating state transitions that are bridged by IsiA and Slr1128. The bound carotenoids, especially zeaxanthin, may function as antioxidants and directly dissipate excessive excitation energy.

## Methods

### Growth conditions and HL treatment

*Synechocystis* cells were cultivated in BG-11 medium with 10 mM TES, pH 8.2, at 30°C. The culture was bubbled with air under low-intensity light (LL; 40 μmol of photon m^−2^ s^−1^) or high-intensity light (HL; 400 μmol of photon m^−2^ s^−1^) conditions. For the experiments conducted under HL, cells in the mid-logarithmic growth phase (OD_730_ ~ 0.8) were diluted with fresh medium to OD_730_ ~ 0.1 and exposed to HL at 30°C.

### Thylakoid membrane preparation and fractionation of membrane protein complexes

Thylakoid membranes were prepared as previously described^43^ with some modifications. Briefly, cell pellets derived from cells grown to the mid-logarithmic phase were resuspended in ice-cold SMN thylakoid buffer (50 mM 3-(N-morpholino)-propanesulfonic acid (pH 7.0), 0.4 M sucrose, 10 mM NaCl, 5 mM MgCl_2_, and 1 mM freshly made phenylmethylsulfonyl fluoride). An equal volume of glass beads pre-wetted with thylakoid buffer was added to the cell suspension, and the cells were broken in a Bead-Beater with an ice-jacketed sample chamber in six breakage cycles at full speed (30 s of burst, followed by 5 min of chilling). The homogenate was centrifuged at 1,800 × g for 10 min to remove unbroken cells, cellular debris, and glass beads. The membranes in supernatant were then pelleted by centrifugation at 50,000 x g at 4°C for 60 min. After washing with 2 mM dodecyl maltoside to remove any remaining phycobilisomes, the membranes were washed twice and resuspended in thylakoid buffer to a chlorophyll a concentration of 1 mg/ml. The chlorophyll a concentration was estimated from the dimethylfluoride extract by the previously reported formula^44^:
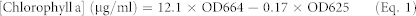


To fractionate membrane protein complexes, 150 μl of 10% dodecyl maltoside was added to the thylakoid membrane to achieve a detergent to chlorophyll ratio of 15:1. The membrane was solubilized at 4°C for 30 min before it was loaded onto a 10–30% (w/w) step sucrose gradient and centrifuged at 160, 000 x g for 16 h at 4°C. Pigmented fractions were collected and stored at −80°C until use.

### Blue-Native, Tricine-PAGE, and immuno-blotting

Blue-Native PAGE was performed as described^45^. For electrophoresis in the second dimension, the single band of the blue native gel was excised and denatured in 1.5 X SDS sample buffer (50 mM Tris-HCl (pH 6.8), 3% SDS, 150 mM DTT, and 0.01% bromophenol blue) for 30 min at room temperature^46^. The gel slice was then laid onto a 12–20% Tricine-SDS gel with 6 M urea as described. The proteins were visualized by silver staining^47^. Polypeptides resolved by SDS-PAGE were electro-transferred onto a PVDF membrane. After the blocking step, the membranes were incubated with polyclonal primary antibodies (Agrisera) followed by horseradish peroxidase-conjugated secondary antibody (Sigma-Aldrich). The reactive bands were detected by enhanced chemiluminescence detection reagents (GE healthcare). The bands were quantified using ImageJ Ver1.36 (National Institutes of Health, USA).

### Database searching

All MS/MS samples were analyzed using Mascot (Matrix Science, London, UK; version2.2.03). Mascot was set up to search the CBInr_061307 database (selected for Bacteria, unknown version, 2419804 entries) assuming the digestion enzyme trypsin. Mascot was searched with a fragment ion mass tolerance of 0.50 Da and a parent ion tolerance of 2.0 Da. The iodoacetamide derivative of cysteine was specified in Mascot as a fixed modification. S-carbamoylmethylcysteine cyclization (N-terminus) of the N-terminus, oxidation of methionine and acetylation of the N-terminus were specified in Mascot as variable modifications.

### Criteria for protein identification

Scaffold (version Scaffold-01_07_00, Proteome Software Inc., Portland, OR) was used to validate MS/MS-based peptide and protein identifications. Peptide identifications were accepted if they could be established at greater than 80.0% probability as specified by the Peptide Prophet algorithm^48^. Protein identifications were accepted if they could be established at greater than 95.0% probability and contained at least three identified peptides. Protein probabilities were assigned by the Protein Prophet algorithm^49^. Proteins that contained similar peptides and could not be differentiated based on MS/MS analysis alone were grouped to satisfy the principles of parsimony.

### SEAD crosslinking

The complex collected from the sucrose gradient was allowed to react in the dark with a 10-fold molar excess of freshly prepared SEAD. The reaction was quenched by adding a 30-fold molar excess of lysine, and the derivatized complex was separated from free reagent by gel filtration through spin chromatography columns (Bio-Rad, Hercules, CA) filled with porous polyacrylamide (Bio-Gel P-2 from Bio-Rad). Photoactivation was carried out for 15 min by exposing the sample to a black-ray, long-wave, 100-W ultraviolet lamp (Ultra Violet Products Inc., San Gabriel, CA). Then 4X Tris-glycine SDS reducing sample buffer was added at a ratio of 1:3 to the reaction mix, and the mix was incubated for 30 min at 22°C and then heated for 5 min at 75°C before electrophoresis.

### Anti-PsaD Immno-Coprecipitation

The complex collected from the sucrose gradient was allowed to react with anti-PsaD (Agrisera) at 0.5–1 mg protein sample/1 μg antibody on ice for 90 min with occasional tube inversion.

The mixture was then added to the SMN-equilibrated Protein G Resin (Sigma-Aldrich), and allowed to react on ice for 1 h with occasional inversion. The Protein G Resin was loaded on a column and washed with 5X bed volume of SMN with 0.1% n-dodecyl-beta-D-maltoside (DM, Sigma-Aldrich). The protein complex was then eluted with elution buffer (Citri acid 0.1 M, pH 2.0) and immediately neutralized with 1 M Tris-HCl (pH 8.5 to pH 7.4), and dialyzed against 20 mM Tris-HCl, pH 7.4. The whole procedure was performed at 4°C and in dim light or in darkness, whenever possible.

### Analysis of Carotenoid Composition by HPLC

Pigments extracted with methanol were subjected to HPLC analysis essentially as described^29^. Pigments were identified by comparing their retention times and absorption spectra^29^.

### Analysis of state transition

Wild-type and mutant cells in the mid-logarithmic growth phase (OD730, 0.6–0.8) were collected and resuspended to an OD730 of 0.6 with fresh BG-11 medium. State transition was analyzed by Chl fluorescence using a Dual-PAM-100 P700 & Chl Fluorescence Measuring System (Heinz Walz, Germany) as described previously^7^. A far-red light was first applied to the dark-adapted cyanobacterial cultures to fully oxidize PSI, which was followed by a series of saturation flashes to determine the S1 level. An actinic light of 100 μmol of photon m^−2^ s^−1^ was subsequently turned on to induce photosynthesis. The S2 level was then assessed by a saturation pulse before the far-red light was applied again to drive the S2-S1 transition.

## Author Contributions

S.D. and J.Z. were responsible for study conception and design, data collection and analysis, manuscript writing and final approval of the manuscript; S.J. and C.H. for data collection and analysis, and final approval of the manuscript; Q.H. and Q.W. for conception and design, critical revision and manuscript writing, and final approval of the manuscript. All authors read and approved the final manuscript.

## References

[b1] Mittler R. Oxidative stress, antioxidants and stress tolerance. Trends in Plant Science 7, 405–410 (2002). 12234732 10.1016/s1360-1385(02)02312-9

[b2] Niyogi K. K. Photoprotection revisited: Genetic and Molecular Approaches. Annual Review of Plant Physiology and Plant Molecular Biology 50, 333–359, 10.1146/annurev.arplant.50.1.333 (1999). 15012213

[b3] Dolganov N. A., Bhaya D. & Grossman A. R. Cyanobacterial protein with similarity to the chlorophyll a/b binding proteins of higher plants: evolution and regulation. Proceedings of the National Academy of Sciences of the United States of America 92, 636–640 (1995). 7831342 10.1073/pnas.92.2.636 PMC42797

[b4] Funk C. & Vermaas W. A Cyanobacterial Gene Family Coding for Single-Helix Proteins Resembling Part of the Light-Harvesting Proteins from Higher Plants. Biochemistry 38, 9397–9404 (1999). 10413515 10.1021/bi990545+

[b5] He Q., Dolganov N., Bjorkman O. & Grossman A. R. The High Light-inducible Polypeptides in Synechocystis PCC6803. Expression and function in high light. J. Biol. Chem. 276, 306–314, 10.1074/jbc.M008686200 (2001). 11024039

[b6] Wang Q. *et al.* The High Light-Inducible Polypeptides Stabilize Trimeric Photosystem I Complex under High Light Conditions in Synechocystis PCC 6803. Plant Physiol. 147, 1239–1250, 10.1104/pp.108.121087 (2008). 18502976 PMC2442545

[b7] Wang Q., Hall C. L., Al-Adami M. Z. & He Q. IsiA Is Required for the Formation of Photosystem I Supercomplexes and for Efficient State Transition in Synechocystis PCC 6803. PLoS ONE 5, e10432 (2010). 20454661 10.1371/journal.pone.0010432 PMC2862709

[b8] Chitnis V. P. *et al.* Targeted Inactivation of the Gene Psal Encoding a Subunit of Photosystem-I of the Cyanobacterium Synechocystis Sp Pcc 6803. J Biol Chem 268, 11678–11684 (1993). 7685019

[b9] Boekema E. J. *et al.* Evidence for a Trimeric Organization of the Photosystem-I Complex from the Thermophilic Cyanobacterium Synechococcus Sp. Febs Letters 217, 283–286 (1987).

[b10] Kruip J., Bald D., Boekema E. & Rogner M. Evidence for the Existence of Trimeric and Monomeric Photosystem-I Complexes in Thylakoid Membranes from Cyanobacteria. Photosynthesis Research 40, 279–286 (1994). 24309946 10.1007/BF00034777

[b11] Tsiotis G., Haase W., Engel A. & Michel H. Isolation and Structural Characterization of Trimeric Cyanobacterial Photosystem-I Complex with the Help of Recombinant Antibody Fragments. European Journal of Biochemistry 231, 823–830 (1995). 7649183 10.1111/j.1432-1033.1995.tb20767.x

[b12] Karapetyan N. V., Dorra D., Schweitzer G., Bezsmertnaya I. N. & Holzwarth A. R. Fluorescence spectroscopy of the longwave chlorophylls in trimeric and monomeric photosystem I core complexes from the cyanobacterium Spirulina platensis. Biochemistry 36, 13830–13837 (1997). 9374860 10.1021/bi970386z

[b13] Shubin V. V., Tsuprun V. L., Bezsmertnaya I. N. & Karapetyan N. V. Trimeric Forms of the Photosystem-I Reaction-Center Complex Pre-Exist in the Membranes of the Cyanobacterium Spirulina-Platensis. Febs Letters 334, 79–82 (1993). 8224233 10.1016/0014-5793(93)81685-s

[b14] Palsson L. O., Dekker J. P., Schlodder E., Monshouwer R. & vanGrondelle R. Polarized site-selective fluorescence spectroscopy of the long-wavelength emitting chlorophylls in isolated Photosystem I particles of Synechococcus elongatus. Photosynthesis Research 48, 239–246 (1996). 24271304 10.1007/BF00041014

[b15] Shubin V. V., Bezsmertnaya I. N. & Karapetyan N. V. Efficient Energy-Transfer from the Long-Wavelength Antenna Chlorophylls to P700 in Photosystem-I Complexes from Spirulina-Platensis. Journal of Photochemistry and Photobiology B-Biology 30, 153–160 (1995).

[b16] Vangrondelle R., Dekker J. P., Gillbro T. & Sundstrom V. Energy-Transfer and Trapping in Photosynthesis. Biochimica Et Biophysica Acta-Bioenergetics 1187, 1–65 (1994).

[b17] Trissl H. W. Long-Wavelength Absorbing Antenna Pigments and Heterogeneous Absorption-Bands Concentrate Excitons and Increase Absorption Cross-Section. Photosynthesis Research 35, 247–263 (1993). 24318755 10.1007/BF00016556

[b18] Wilson A. *et al.* A soluble carotenoid protein involved in phycobilisome-related energy dissipation in cyanobacteria. Plant Cell 18, 992–1007 (2006). 16531492 10.1105/tpc.105.040121 PMC1425857

[b19] Wilson A. *et al.* A photoactive carotenoid protein acting as light intensity sensor. Proceedings of the National Academy of Sciences 105, 12075–12080, 10.1073/pnas.0804636105 (2008). PMC2575289 18687902

[b20] Holt K. T. & Krogmann D. W. A carotenoid-protein from cyanobacteria. Biochimica et Biophysica Acta (BBA) - Bioenergetics 637, 408–414 (1981).

[b21] Kerfeld C. Structure and Function of the Water-Soluble Carotenoid-Binding Proteins of Cyanobacteria. Photosynthesis Research 81, 215–225 (2004). 16034528 10.1023/B:PRES.0000036886.60187.c8

[b22] Polívka T., Kerfeld C. A., Pascher T. & Sundstrom V. Spectroscopic Properties of the Carotenoid 3'-Hydroxyechinenone in the Orange Carotenoid Protein from the Cyanobacterium Arthrospira maxima. Biochemistry 44, 3994–4003 (2005). 15751975 10.1021/bi047473t

[b23] Wu Y. P. & Krogmann D. W. The orange carotenoid protein of Synechocystis PCC 6803. Biochimica et Biophysica Acta (BBA) - Bioenergetics 1322, 1–7 (1997). 10.1016/s0005-2728(97)00067-4 9398074

[b24] Kerfeld C. A. Water-soluble carotenoid proteins of cyanobacteria. Archives of Biochemistry and Biophysics 430, 2–9 (2004). 15325905 10.1016/j.abb.2004.03.018

[b25] Divers-Pierluissi M. & Krogmann D. W. A zeaxanthin protein from Anacystis nidulans. Biochimica et Biophysica Acta (BBA) - Bioenergetics 933, 372–377 (1988).

[b26] Kerfeld C. A. *et al.* The Crystal Structure of a Cyanobacterial Water-Soluble Carotenoid Binding Protein. Structure 11, 55–65 (2003). 12517340 10.1016/s0969-2126(02)00936-x

[b27] Sun J., Ke A., Jin P., Chitnis V. P. & Chitnis P. R. Isolation and functional study of photosystem I subunits in the cyanobacterium Synechocystis sp. PCC 6803. Methods in enzymology 297, 124–139 (1998). 9750206 10.1016/s0076-6879(98)97010-0

[b28] Chitnis V. P. & Chitnis P. R. Psal Subunit Is Required for the Formation of Photosystem-I Trimers in the Cyanobacterium Synechocystis Sp Pcc-6803. Febs Letters 336, 330–334 (1993). 8262256 10.1016/0014-5793(93)80831-e

[b29] Takaichi S., Maoka T. & Masamoto K. Myxoxanthophyll in Synechocystis sp. PCC 6803 is Myxol 2′-Dimethyl-Fucoside, (3R,2′S)-Myxol 2′-(2,4-di-O-Methyl-*α*-l-Fucoside), not Rhamnoside. Plant and Cell Physiology 42, 756–762, 10.1093/pcp/pce098 (2001). 11479383

[b30] Campbell D., Hurry V., Clarke A. K., Gustafsson P. & Oquist G. Chlorophyll Fluorescence Analysis of Cyanobacterial Photosynthesis and Acclimation. Microbiol. Mol. Biol. Rev. 62, 667–683 (1998). 9729605 10.1128/mmbr.62.3.667-683.1998 PMC98930

[b31] Campbell D. & Oquist G. Predicting Light Acclimation in Cyanobacteria from Nonphotochemical Quenching of Photosystem II Fluorescence, Which Reflects State Transitions in These Organisms. Plant Physiol. 111, 1293–1298, 10.1104/pp.111.4.1293 (1996). 12226362 PMC161011

[b32] Herranen M. *et al.* Towards Functional Proteomics of Membrane Protein Complexes in Synechocystis sp. PCC 6803. Plant Physiol. 134, 470–481, 10.1104/pp.103.032326 (2004). 14730074 PMC316326

[b33] Zilber A. L. & Malkin R. Organization and Topology of Photosystem I Subunits. Plant Physiol. 99, 901–911, 10.1104/pp.99.3.901 (1992). 16669018 PMC1080562

[b34] Chitnis P. R., Reilly P. A. & Nelson N. Insertional inactivation of the gene encoding subunit II of photosystem I from the cyanobacterium Synechocystis sp. PCC 6803. J. Biol. Chem. 264, 18381–18385 (1989). 2509457

[b35] Chitnis V. P. & Chitnis P. R. PsaL subunit is required for the formation of photosystem I trimers in the cyanobacterium Synechocystis sp. PCC 6803. FEBS letters 336, 330–334 (1993). 8262256 10.1016/0014-5793(93)80831-e

[b36] Chitnis V. P., Jung Y.-S., Albee L., Golbeck J. H. & Chitnis P. R. Mutational Analysis of Photosystem I Polypeptides. J. Biol. Chem. 271, 11772–11780, 10.1074/jbc.271.20.11772 (1996). 8662633

[b37] Bottin H. & Lagoutte B. Ferrodoxin and flavodoxin from the cyanobacterium Synechocystis sp PCC 6803. Biochimica et Biophysica Acta (BBA) - Protein Structure and Molecular Enzymology 1101, 48–56 (1992). 10.1016/0167-4838(92)90465-p 1633177

[b38] Giuliana Z. & Giuliana M. Interaction between photosystem I and ferredoxin. European Journal of Biochemistry 169, 143–146 (1987). 2824198 10.1111/j.1432-1033.1987.tb13591.x

[b39] Lagoutte B., Hanley J. & Bottin H. Multiple Functions for the C Terminus of the PsaD Subunit in the Cyanobacterial Photosystem I Complex. Plant Physiol. 126, 307–316, 10.1104/pp.126.1.307 (2001). 11351094 PMC102305

[b40] Wynn R. M., Omaha J. & Malkin R. Structural and functional properties of the cyanobacterial photosystem I complex. Biochemistry 28, 5554–5560, 10.1021/bi00439a032 (2002). 2505837

[b41] Zilber A. L. & Malkin R. Ferredoxin Cross-Links to a 22 kD Subunit of Photosystem I. Plant Physiol. 88, 810–814, 10.1104/pp.88.3.810 (1988). 16666389 PMC1055666

[b42] Pfannschmidt T. Chloroplast redox signals: how photosynthesis controls its own genes. Trends in Plant Science 8, 33–41 (2003). 12523998 10.1016/s1360-1385(02)00005-5

[b43] Shen G. & Vermaas W. F. Chlorophyll in a Synechocystis sp. PCC 6803 mutant without photosystem I and photosystem II core complexes. Evidence for peripheral antenna chlorophylls in cyanobacteria. The Journal of biological chemistry 269, 13904–13910 (1994). 8188669

[b44] Moran R. Formulas for Determination of Chlorophyllous Pigments Extracted with N,N-Dimethylformamide. Plant Physiol 69, 1376–1381 (1982). 16662407 10.1104/pp.69.6.1376 PMC426422

[b45] Schagger H. & Vonjagow G. Blue Native Electrophoresis for Isolation of Membrane-Protein Complexes in Enzymatically Active Form. Analytical Biochemistry 199, 223–231 (1991). 1812789 10.1016/0003-2697(91)90094-a

[b46] Schagger H. Tricine-SDS-PAGE. Nature Protocols 1, 16–22 (2006). 10.1038/nprot.2006.4 17406207

[b47] Rabilloud T., Carpentier G. & Tarroux P. Improvement and Simplification of Low-Background Silver Staining of Proteins by Using Sodium Dithionite. Electrophoresis 9, 288–291 (1988). 2466660 10.1002/elps.1150090608

[b48] Keller A., Nesvizhskii A. I., Kolker E. & Aebersold R. Empirical Statistical Model To Estimate the Accuracy of Peptide Identifications Made by MS/MS and Database Search. Analytical Chemistry 74, 5383–5392, 10.1021/ac025747h (2002). 12403597

[b49] Nesvizhskii A. I., Keller A., Kolker E. & Aebersold R. A Statistical Model for Identifying Proteins by Tandem Mass Spectrometry. Analytical Chemistry 75, 4646–4658 (2003). 14632076 10.1021/ac0341261

